# Elucidation of the Molecular Pathways Involved in the Protective Effects of AUY-922 in LPS-Induced Inflammation in Mouse Lungs

**DOI:** 10.3390/ph14060522

**Published:** 2021-05-29

**Authors:** Mohammad S. Akhter, Mohammad A. Uddin, Khadeja-Tul Kubra, Nektarios Barabutis

**Affiliations:** School of Basic Pharmaceutical and Toxicological Sciences, College of Pharmacy, University of Louisiana Monroe, Monroe, LA 71201, USA; akhterms@warhawks.ulm.edu (M.S.A.); uddinma@warhawks.ulm.edu (M.A.U.); kubrak@warhawks.ulm.edu (K.-T.K.)

**Keywords:** acute lung injury, acute respiratory distress syndrome, Hsp90 inhibitor, P53, unfolded protein response

## Abstract

Acute lung injury (ALI) and acute respiratory distress syndrome (ARDS) cause thousands of deaths every year and are associated with high mortality rates (~40%) due to the lack of efficient therapies. Understanding the molecular mechanisms associated with those diseases will most probably lead to novel therapeutics. In the present study, we investigated the effects of the Hsp90 inhibitor AUY-922 in the major inflammatory pathways of mouse lungs. Mice were treated with LPS (1.6 mg/kg) via intratracheal instillation for 24 h and were then post-treated intraperitoneally with AUY-922 (10 mg/kg). The animals were examined 48 h after AUY-922 injection. LPS activated the TLR4-mediated signaling pathways, which in turn induced the release of different inflammatory cytokines and chemokines. AUY-922 suppressed the LPS-induced inflammation by inhibiting major pro-inflammatory pathways (e.g., JAK2/STAT3, MAPKs), and downregulated the IL-1β, IL-6, MCP-1 and TNFα. The expression levels of the redox regulator APE1/Ref1, as well as the DNA-damage inducible kinases ATM and ATR, were also increased after LPS treatment. Those effects were counteracted by AUY-922. Interestingly, this Hsp90 inhibitor abolished the LPS-induced pIRE1α suppression, a major component of the unfolded protein response. Our study elucidates the molecular pathways involved in the progression of murine inflammation and supports our efforts on the development of new therapeutics against lung inflammatory diseases and sepsis.

## 1. Introduction

Human lungs encounter different atmospheric insults, as well as toxic molecules circulating in the blood. Lung injury can be direct (pulmonary or primary) or indirect (extrapulmonary or secondary). Direct lung injury causes local damage to the lung epithelium, while indirect injury results in damage to the lung endothelium [1]. Both direct and indirect lung injuries trigger inflammation in lung epithelial and endothelial cells, leading to acute lung injury (ALI) and acute respiratory distress syndrome (ARDS).

The pulmonary vasculature is a mono-layered, non-fenestrated endothelial cell lining, which selectively permits the transport of fluids and solutes between the vasculature and the interstitium. The intercellular interactions among endothelial cells through tight and adherens junctions dictate the integrity of the vascular barrier. The epithelium layer forms a very tight barrier, which prohibits the transfer of small molecules to the alveolar space but allows the oxygen and carbon dioxide exchange [2]. In normal physiological conditions, both the alveolar type I and II cells drain out excess fluids from the alveolar airspace to the interstitium through apical sodium channels and basolateral Na^+^/K^+^-ATPase. The excess interstitial fluid is cleared by the lung lymphatics and microcirculation. However, the alveolar fluid clearance (AFC) is impaired by hypoxia, hypercapnia, elevated lung vascular pressure, pathogens (e.g., influenza), as well as several cytokines including interleukin 1-β (IL-1β), interleukin-8 (IL-8) and transforming growth factor-β (TGF-β) [3].

ARDS is a syndrome of acute respiratory failure associated with arterial hypoxemia and dyspnea. Increased endothelial permeability is the hallmark of ARDS. Lung endothelial cells are key regulators of inflammatory responses. Pathogens, bacterial toxins (e.g., lipopolysaccharides) and sepsis activate the lung endothelial cells. Those activated cells increase the expression of the leukocyte adhesion molecules including intracellular adhesion molecule-1 (ICAM-1), vascular cell adhesion molecule-1 (VCAM-1) and E-selectin [4]. Those events result in the accumulation of neutrophil-platelet aggregates in the lung microvessels. Neutrophils and platelets are involved in the regulation of the lung vascular hyper-permeability.

Reactive oxygen species (ROS), reactive nitrogen species (RNS), tumor necrosis factor (TNF), IL-1β, IL-8, angiopoietin 2, platelet-activating factor (PAF) and monocyte chemoattractant protein-1 (MCP-1) contribute to the endothelial barrier dysfunction. Hence, proteinous fluid, neutrophils and erythrocytes enter the alveolar interstitium, causing edema. In addition, the AFC mechanism of the lung epithelium is severely compromised due to inflammation-associated epithelial cell damage. The rate of pulmonary edema resolution is markedly impaired in ARDS patients. Inflammation exerts a critical role in the pathogenesis of different lung diseases including chronic obstructive pulmonary disease (COPD), asthma, pneumonia, idiopathic pulmonary fibrosis (IPF) [5] and bronchitis.

The unfolded protein response (UPR) is a highly conserved endoplasmic reticulum (ER) surveillance mechanism that ensures the integrity of the folded proteins and regulates the protein folding capacity of the ER. A diverse variety of physiological conditions (e.g., cancer, immunological disorders, neurodegenerative diseases, pathogens) challenge the ER homeostasis, causing the accumulation of misfolded or unfolded proteins in the ER lumen. When the levels of these misfolded or unfolded proteins surpass a certain threshold, it triggers the activation of UPR. This evolutionary signal transduction pathway (UPR) restricts gene transcription and mRNA translation. It also activates the ER-associated protein degradation (ERAD) pathway to reduce the burden of unfolded or misfolded proteins in the ER lumen. Three transmembrane protein receptors, namely protein kinase RNA like ER kinase (PERK), activating transcription factor 6 (ATF6) and inositol-requiring enzyme 1 (IRE1), comprise UPR and sense the status of the misfolded or unfolded proteins. Those sensors transmit the information to the nucleus and cytosol to adjust the protein folding capacity of the ER or, in severe conditions, to induce cell death.

UPR is associated with autophagy [6,7], cell cycle progression, cellular secretion [7] and inflammation [8,9]. Recent data suggest that a mild induction of UPR has protective effects against inflammation-induced lung endothelial barrier dysfunctions [10]. The UPR suppressor Kifunensine induces the permeability of both human and bovine lung endothelial cells and increases the activation of myosin light chain 2 (MLC2) and cofilin [11]. It was recently revealed that UPR mediates the barrier-enhancing effects of the growth hormone-releasing hormone (GHRH) antagonists [12,13,14].

Heat shock protein 90 (Hsp90) is a molecular chaperone responsible for the folding and maturation of a plethora of proteins, including steroid hormone receptors and kinases [15]. It is associated with cancer progression, multidrug resistance and metastasis. Therefore, different Hsp90 inhibitors have been developed to treat malignancies [16]. Hsp90 inhibitors suppress the tyrosine phosphorylation of Hsp90 in vivo and in vitro [17]. That molecular chaperone is also associated with the stability of many kinases of the MAPK family including three major families—extracellular signal regulated kinases (ERKs), c-Jun NH_2_-terminal kinases (JNKs) and p38/ SAPKs (stress-activated protein kinases). Those kinases are involved in various cellular processes such as inflammation, cell proliferation, stress response, survival and migration. The inhibition of Hsp90 by geldanamycin and AUY-922 significantly downregulates MAPKs and their upstream components in cancer cells [18,19]. Those compounds suppress the inflammatory pathway JAK2/STAT3 [20] since JAK2 is an Hsp90 client protein [21]. AUY-922 was shown to suppress the redox regulator APE1/Ref1 through the induction of P53 and to support lung endothelial barrier function [22]. AUY-922, 17-AAG and 17-DMAG activate the UPR sensors ATF6, IRE1α and PERK. These events occur in both bovine and human lung endothelial cells, as well as in mice [23,24].

The nuclear factor-κB (NF-κB) is a key transcription factor, required for the induction of a large number of inflammatory genes, including those encoding for IL-1β, IL-6, IL-12p40, TNF-α, MCP-1 and cyclooxygenase-2 [25]. Hsp90 inhibitors suppress the NF-κB-mediated transcription, and therefore, inhibit the RNA expression of different inflammatory markers including IL-1β, IL-6, TNF-α and MCP-1. In lung endothelial cells, type 2 sirtuin (Sirt-2) binds to the NF- κB target gene promoter to block the recruitment and transcription of NF-κB. During inflammatory events, Sirt-2 dissociates from the promoter region which facilitates the NF-κB—targeted gene transcription. 17-AAG stabilizes the Sirt-2/promoter interaction and inhibits the NF-κB-associated gene transcription [26]. Other studies have reported that Hsp90 inhibitors cause the dissociation of the inhibitor of IκB kinase (IKK) complex, which results in NF-κB deactivation. AUY-922, geldanamycin, 17-AAG, 17-DMAG and PU-H71 have previously prevented the nuclear translocation of NF-κB [27]. The current study investigates the effects of AUY-922 in major inflammatory pathways and the UPR sensor IRE1α in a murine model of ALI.

## 2. Results

### 2.1. AUY-922 Inhibits the Activation of ERK1/2 Pathway by LPS

**Figure 1:**
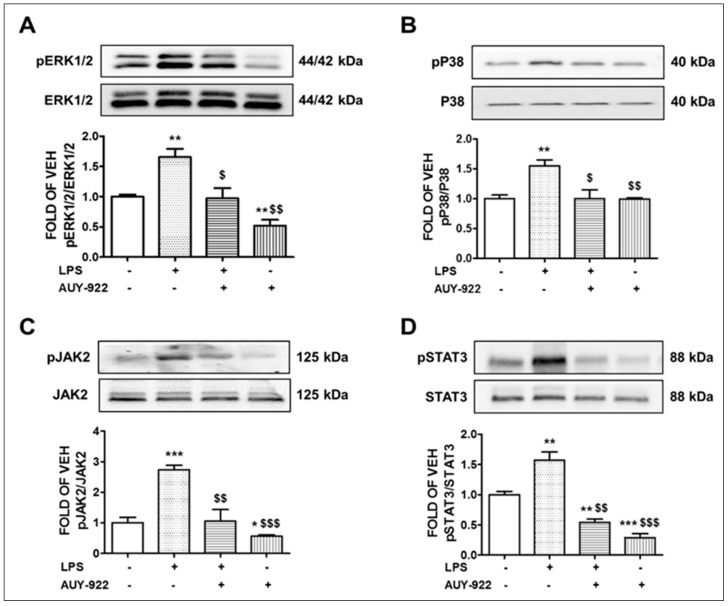
Effects of LPS and AUY-922 on pERK1/2, pP38, pJAK2 and pSTAT3 expression in mouse lungs. Western blot analysis of phosphorylated ERK1/2 (pERK1/2) and ERK1/2 (**A**), phosphorylated P38 (pP38) and P38 (**B**), phosphorylated JAK2 (pJAK2) and JAK2 (**C**), phosphorylated STAT3 (pSTAT3) and STAT3 (**D**) expression in lungs retrieved from mice 72 h after intratracheal injection of either vehicle (saline) or LPS (1.6 mg/kg) and post-treated (24 h after LPS) with an intraperitoneal injection of either AUY-922 (10 mg/kg each, dissolved in 10% DMSO) or vehicle (10% DMSO in saline). The signal intensity of protein bands was analyzed by densitometry. Protein levels of pERK1/2, pP38, pJAK2 and pSTAT3 were normalized to ERK1/2, P38, JAK2 and STAT3, respectively. * *p* < 0.05, ** *p* < 0.01, *** *p* < 0.001 vs. vehicle (VEH) and ^$^ *p* < 0.05, ^$$^ *p* < 0.01, ^$$$^ *p* < 0.001 vs. LPS, *n* = 3 animals per group. Means ± SEM.—indicates the absence, and + indicates the presence of the corresponding reagent in the treatments.

ERK1/2 is a member of the MAPK family, and it is activated through phosphorylation. Activation of ERK1/2 promotes the IL-1β -mediated pro-inflammatory IL-6 expression [28] and has been involved in chronic states of inflammation. Animals treated with LPS (1.6 mg/kg) for 24 h showed an elevation of the phosphorylated ERK1/2 expression levels in lung tissues as compared to the vehicle-treated mice. However, this activation was significantly decreased after AUY-922 treatment for 48 h (10 mg/kg). Mice treated with AUY-922 exerted a significant suppression of the ERK1/2 phosphorylation compared to the control group. The results are demonstrated in Figure 1A.

### 2.2. AUY-922 Counteracts the LPS-Induced Phosphorylation of the P38 MAPK Pathway

P38 kinases are activated in macrophages and endothelial cells by LPS and TNFα [29,30,31]. The phosphorylation of P38 MAPK causes its activation. Our results demonstrate that LPS treatment for 24 h at the dose of 1.6 mg/kg induced the phosphorylation or activation of the P38 MAPK pathway in the mouse lungs. Conversely, the AUY-922 (10 mg/kg)—treated mice exhibited a reduced activation of P38 compared to the LPS-treated mice. Mice treated with LPS for 24 h and post-treated with AUY-922 for 48 h exerted lower levels of phosphorylated P38 MAPK. That indicates the protective effects of AUY-922 against LPS-induced P38 MAPK activation. The results appear in Figure 1B.

### 2.3. AUY-922 Suppresses the LPS-Induced Activation of JAK2/STAT3 Pathway

The JAK2/STAT3 signaling pathway is involved in various biological processes including inflammation. The phosphorylation of STAT3 at Tyr705 and Ser727 is mediated by the receptor-associated tyrosine kinase JAK2, and both molecules are dephosphorylated by AUY-922 [32]. Our results (Figure 1C,D) suggest that the pro-inflammatory JAK2/STAT3 pathway was activated due to LPS treatment in the mouse lungs. Those effects were significantly suppressed in mice post-treated with AUY-922 for 48 h. The AUY-922-treated group that was not exposed to LPS showed a decrease in the activation of the JAK2/STAT3 pathway, as compared to the control and LPS-treated groups. Our results demonstrate that phospho (p)-JAK2 (Figure 1C) and phospho (p)-STAT3 (Figure 1D) levels were increased in the LPS-treated mice as compared to the vehicle (saline)-treated mice. The Hsp90 inhibitor opposed those events.

### 2.4. AUY-922 Counteracts the LPS-Induced IL-1β, IL-6, MCP-1 and TNF-α Expression

**Figure 2:**
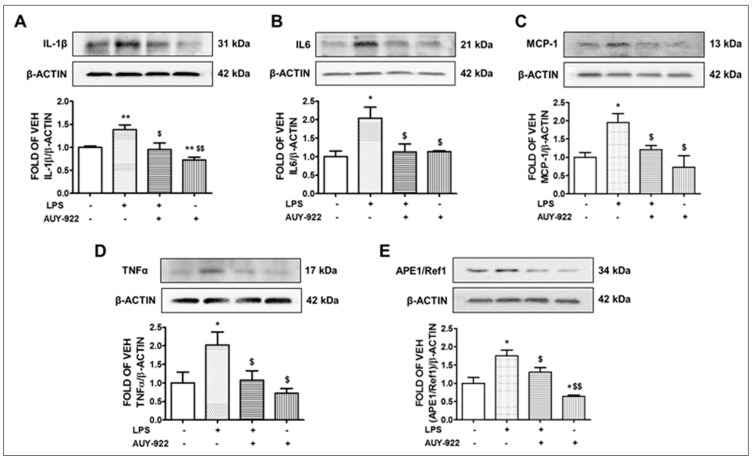
Effects of LPS and AUY-922 on IL-1β, IL6, MCP-1, TNFα and APE1/Ref1 expression in mouse lungs. Western blot analysis of IL-1β and β-actin (**A**), IL6 and β-actin (**B**), MCP-1 and β-actin (**C**), TNFα and β-actin (**D**), APE1/Ref1 and β-actin (**E**) expression in lungs retrieved from mice 72 h after intratracheal injection of either vehicle (saline) or LPS (1.6 mg/kg) and post-treated (24 h after LPS) with an intraperitoneal injection of either AUY-922 (10 mg/kg each, dissolved in 10% DMSO) or vehicle (10% DMSO in saline). The signal intensity of protein bands was analyzed by densitometry. Protein levels were normalized to β-actin. * *p* < 0.05, ** *p* < 0.01 vs. vehicle (VEH) and ^$^ *p* < 0.05, ^$$^ *p* < 0.01 vs. LPS, *n* = 3 animals per group. Means ± SEM.—indicates the absence, and + indicates the presence of the corresponding reagent in the treatments.

Cytokines and chemokines promote inflammation. The wild type C57BL/6 mice were subjected to LPS treatment for 24 h at the dose of 1.6 mg/kg, followed by post-treatment with AUY-922 (10 mg/kg) for 48 h. Our results demonstrate that LPS induced the expression of IL-1β (Figure 2A), IL-6 (Figure 2B), TNF-α (Figure 2D) and chemokine MCP-1 (Figure 2C) in the lungs. On the other hand, AUY-922 treatment significantly inhibited the LPS-induced expression of those cytokines and chemokines. Moreover, in the group of mice treated with AUY-922 (no LPS treatment), the expression levels of IL-1β were significantly reduced as compared to the control group.

### 2.5. LPS Induces the Expression Levels of APE1/Ref1 and AUY-922 Exerts the Opposite Effects

Apurinic/apyrimidinic endonuclease 1/ redox effector factor 1 (APE1/Ref1) is the upstream effector of vascular endothelial growth factor (VEGF), and it is strongly involved in the pathogenesis of various inflammatory diseases. This transcription factor regulates the expression of different immune responses and inflammatory mediators, including NF-kB, via a redox-based mechanism [33]. Mice treated with LPS for 24 h showed an increased level of APE1/Ref1 expression in the lungs. That effect may be due to the upregulation of oxidative stress and other inflammatory mediators by LPS. The induction of APE1/Ref1 by LPS was 1.7-fold higher than the control group. However, treatment with AUY-922 (10 mg/kg) for 48 h significantly suppressed the protein expression of APE1/Ref1. AUY-922 significantly counteracted the APE1/Ref1 levels in the LPS-treated mice (Figure 2E).

### 2.6. AUY-922 Suppresses the Levels of the LPS-Induced ATR Phosphorylation

**Figure 3:**
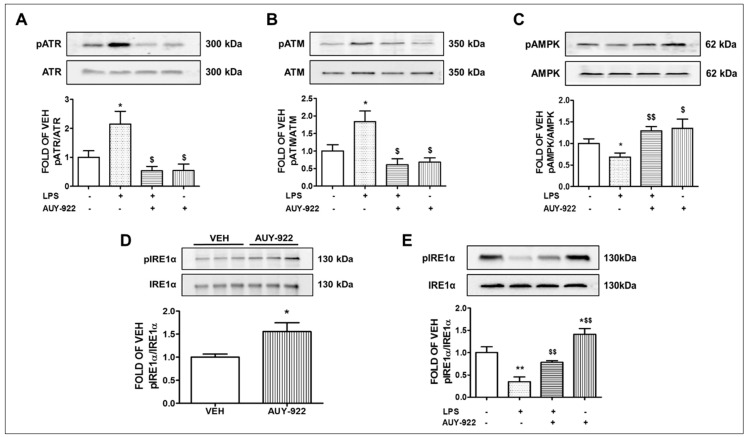
Effects of hsp90 inhibition in major inflammatory cascades of the murine inflamed lungs. Western blot analysis of phosphorylated ATR (pATR) and ATR (**A**), phosphorylated ATM (pATM) and ATM (**B**), phosphorylated AMPK (pAMPK) and AMPK (**C**), phosphorylated IRE1α (pIRE1α) and IRE1α (**E**) expression in lungs retrieved from mice 72 h after intratracheal injection of either vehicle (saline) or LPS (1.6 mg/kg) and post-treated (24 h after LPS) with an intraperitoneal injection of either AUY-922 (10 mg/kg each, dissolved in 10% DMSO) or vehicle (10% DMSO in saline). The signal intensity of protein bands was analyzed by densitometry. Protein levels of pATR, pATM, pAMPK and pIRE1α were normalized to ATR, ATM, AMPK and IRE1α, respectively. * *p* < 0.05, ** *p* < 0.01 vs. vehicle (VEH) and ^$^ *p* < 0.05, ^$$^ *p* < 0.01 vs. LPS, *n* = 3 animals per group. Means ± SEM. Western Blot analysis of phosphorylated IRE1α (pIRE1α) and IRE1α (**D**) in mouse lungs treated with either vehicle (10% DMSO in saline) or AUY-922 (10 mg/kg each, dissolved in 10% DMSO) via an intraperitoneal injection for 48 h. The signal intensity of pIRE1α and IRE1α was analyzed by densitometry. Protein levels of pIRE1α were normalized to IRE1α. * *p* < 0.05 vs. vehicle (VEH), number of animals in each group = 3. Means ± SEM.—indicates the absence, and + indicates the presence of the corresponding reagent in the treatments.

Our previous observations revealed that the anti-inflammatory and barrier-enhancing effects of Hsp90 inhibitors are associated with the induction of P53. Several studies reported the degradation of P53 due to its phosphorylation [34]. This protein is also the downstream target of DNA-damage inducible kinases ATM and ATR. ATR phosphorylation activates different cell cycle checkpoint kinases. The expression levels of phosphorylated ATR in the LPS-treated mice group is 2.1-fold higher compared to its littermate control group. Those levels were significantly reduced after treatment with AUY-922 for 48 h. AUY-922 substantially suppressed the ATR phosphorylation. The results appear in Figure 3A.

### 2.7. AUY-922 Reduces the Expression Levels of the Phosphorylated ATM

DNA damage activates ATM kinases [35]. Phosphorylation of ATM induces its activity, which in turn phosphorylates MDM2. The LPS-treated mice exhibited higher levels (1.8-fold) of phosphorylated ATM in their lungs. However, treatment with the Hsp90 inhibitor AUY-922 for 48 h suppressed the active ATM. AUY-922 post-treatment counteracted the LPS-induced ATM phosphorylation (Figure 3B).

### 2.8. AUY-922 Counteracts the LPS-Induced Suppression of pAMPK

The AMP-activated protein kinase (AMPK) is involved in the regulation of various important physiological functions, such as endothelial cell energy supply and endothelial NOS activation [36], and induces P53 [36,37]. Hence, phosphorylation of AMPK supports endothelial integrity and represents a promising strategy for the protection of the vascular endothelial barrier function. In this study, mice treated with LPS for 24 h showed a significant reduction in the phosphorylation and activation of AMPK. The LPS-induced suppression of AMPK was counteracted by the AUY-922 treatment (48 h) (Figure 3C).

### 2.9. AUY-922 Suppresses the LPS-Induced Deactivation of IRE1α

IRE1α is the most conserved sensor of UPR signaling. Upon dissociation from BiP, it homodimerizes and autophosphorylates to trigger downstream signaling mechanisms. Mice treated with AUY-922 for 48 h exerted increased levels of phosphorylated IRE1α (activated form) expression as compared to the vehicle-treated group (Figure 3D). Moreover, LPS suppressed the phosphorylation of IRE1α, an effect opposed by AUY-922 (Figure 3E).

### 2.10. Effects of AUY-922 on LPS-Induced Body Weight Reduction and BALF Protein Concentration

**Figure 4:**
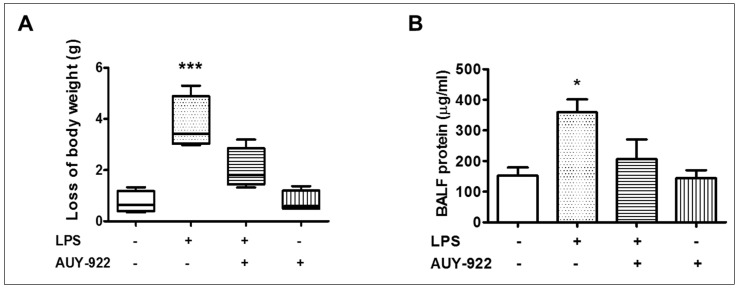
Effects of LPS and hsp90 inhibition on body weight and BALF total protein levels in mice. Male C57BL/6 mice were treated with an intratracheal injection of either vehicle (saline) or LPS (1.6 mg/kg) and post-treated (24 h after LPS) with an intraperitoneal injection of either AUY-922 (10 mg/kg each, dissolved in 10% DMSO) or vehicle (10% DMSO in saline). (**A**) Changes in body weight were determined by weighing the mice before and after each treatment period. (**B**) Measurements of total protein levels in the bronchoalveolar lavage fluid (BALF) of mice. * *p* < 0.05, *** *p* < 0.001 vs. vehicle (VEH), *n* = 4 animals per group. Means ± SEM.—indicates the absence, and + indicates the presence of the corresponding reagent in the treatments.

The changes in body weight and BALF protein concentration after LPS and AUY-922 treatment were observed. LPS treatment for 24 h reduced body weight as compared to the vehicle-treated group. On the other hand, treatment with AUY-922 (10 mg/kg) for 48 h suppressed the LPS-induced loss of body weight (Figure 4A). The BALF protein concentration in the LPS-treated group was significantly increased. Treatment with AUY-922 (48 h) reduced the BALF protein concentration (Figure 4B).

## 3. Discussion

Hsp90 inhibitors are promising anti-inflammatory therapeutic agents due to their ability to target various signaling mechanisms. Hence, several generations of Hsp90 inhibitors have been developed. AUY-922 is a highly specific synthetic resorcinylic isoxazole amide which demonstrates low-nanomolar affinities for the cytosolic isoform of Hsp90 [38]. It demonstrates improved bioavailability, safety and activity profile compared to the earlier Hsp90 inhibitors [39]. It targets the ATP-nucleotide pockets at the N-terminal domain of Hsp90 and blocks the cycling between ADP- and ATP-bound conformations, impairing the chaperoning activities of Hsp90 [40]. 17-AAG belongs to the benzoquinone ansamycin class of Hsp90 inhibitors and significantly suppresses the function of Hsp90. However, it has relatively poor solubility and oral bioavailability.

AUY-922 protects against nitrogen mustard-induced pulmonary fibrosis (PF) and lung dysfunction. Acute exposure to nitrogen mustard is involved in the development of chronic lung injury. AUY-922 decreases the accumulation of extracellular matrix proteins and reduces the histologic evidence of fibrosis in the lungs [41]. 17-AAG suppresses the endothelial permeability by disrupting RhoA signaling. Activated RhoA stimulates its downstream effector Rho kinase (ROCK), which phosphorylates the myosin light chain 2 (MLC2), leading to endothelial hyper-permeability [42]. The expression levels of VE-cadherin and β-catenin indicate the integrity of the endothelial barrier. The transforming growth factor-β1 (TGF-β1) decreases the expression of those adherens junction proteins in bovine lung endothelial cells, indicating a compromised endothelial barrier. Hsp90 inhibition restores the adherens junction protein and prevents the TGF-β1-induced actin stress fiber formation and depolymerization of peripheral microtubules [43]. Hence, Hsp90 inhibitors modulate different signaling pathways in order to protect the lung endothelial barrier.

P53 functions as an orchestrator of anti-inflammatory signaling. It suppresses the redox regulator APE1/Ref1 [22,44,45], inhibits the RhoA/MLC2 pathway [46] and deactivates the actin severing activity of cofilin in endothelial cells [47,48]. P53 is subjected to phosphorylation and subsequent degradation by bacterial toxins such as lipopolysaccharide [49] and lipoteichoic acid [50]. Inhibition of Hsp90 increases P53 expression levels by suppressing its phosphorylation in Ser6, Ser15, Ser33 and Ser392 [49]. Induction of UPR results in increased P53, while UPR suppression reduces P53 levels [51]. That demonstrates that P53 mediates the UPR functions against endothelial barrier hyper-permeability.

The mitogen-activated protein kinase (MAPK) pathway is associated with the activation of endothelial cells in response to numerous inflammatory stimuli [52]. MAPKs are crucial in the modulation of barrier function. Activation of toll-like receptor (TLR) induces the phosphorylation of MAPKs and suppresses the TJ protein expression in brain endothelial cells, causing barrier dysfunction [53]. However, suppression of the MAPK signaling attenuates endothelial activation and downregulates the TNFα-induced expression of VCAM-1 and ICAM-1 [54]. Moreover, Angiotensin II (Ang II), a well-known vasoconstrictor, enhances endothelial permeability by increasing the production of prostaglandins and vascular endothelial growth factor (VEGF). It also activates the extracellular signal-regulated kinase1/2 (ERK1/2), c-Jun NH_2_-terminal kinase 1/2 (JNK1/2) and p38 MAPK. It contributes to the actin cytoskeleton remodeling and stress fiber formation. Inhibition of Ang II increases the endothelial integrity by suppressing the MAPK pathway [55]. Another study reported a direct relationship between MAPK activation, microtubule disassembly and lung endothelial barrier failure [56].

In line with those observations, we revealed that the intra-tracheal administration of LPS induces the activation of ERK1/2 and P38 in mouse lung tissues. LPS is the major component of the outer membrane of gram-negative bacteria and a key pathogenic stimulator of inflammatory signaling. LPS is recognized by the toll-like receptor 4 (TLR4), which is widely expressed in different types of cells including endothelial cells [57], macrophages [58] and lung epithelial cells [59]. Activation of TLR4 stimulates the phosphorylation and activation of MAPKs and forms a complex with myeloid differentiation protein 2 (MD2) to activate myeloid differentiation factor 88 (MyD88). The TLR4-MD2-LPS complex triggers the phosphorylation of protein kinase B (PKB), which activates the nuclear factor-κB (NF- κB) signaling to facilitate inflammatory responses [58]. In this study, we observed a significant induction of different inflammatory cytokines and chemokines such as MCP-1, IL-6 and TNFα in the lung tissues after the LPS treatment. AUY-922 protected against the severity of LPS-induced inflammation by suppressing the MAPKs (Figure 1A,B), IL-1β (Figure 2A), IL-6 (Figure 2B), MCP-1 (Figure 2C) and TNFα (Figure 2D). Similarly, previous studies reported that LPS-treated mice expressed higher levels of IL-2 and IL-10 cytokines in the bronchoalveolar lavage fluid (BALF). AUY-922 counteracted the LPS-induced upregulation of those cytokines [49]. Histopathological analysis of LPS-treated lungs suggested a strong inflammatory response that was counteracted by the Hsp90 inhibitor radicicol [60].

The Janus kinase 2/signal transducer and activator of transcription 3 (JAK2/STAT3) is a signal transduction pathway involved in regulating a range of cellular functions including cell survival, cell-cycle progression, proliferation, angiogenesis and inflammation. JAK2 is a non-receptor tyrosine kinase (nRTK) that phosphorylates STAT3 and causes its dimerization through the SH2 domain and the subsequent translocation to the nucleus. STAT3 can also be phosphorylated by multiple upstream molecules, such as receptor tyrosine kinases, vascular endothelial growth factor receptor (VEGFR) and epidermal growth factor receptor (EGFR). It is also affected by other nRTKs including the Src-family kinases (Src, Fyn, Lyn, etc.) and PI3K [61]. The activation of JAK2/STAT3 pathway mediates endothelial barrier dysfunction by inducing IL-6. This pathway also downregulates the expression of VE-cadherin and tight junction proteins (such as zonula occluden-1) [62]. It also induces VEGF production [63]. Hence, inhibition of STAT3 phosphorylation (mediated by JAK2) exerts anti-inflammatory activities and reduces endothelial permeability. Hsp90 is associated with the IL-6-induced STAT3 signaling at elevated temperatures. Geldanamycin markedly inhibits STAT3 signaling stimulated by IL-6 [64], and other studies reported a correlation between MAPK and JAK2/STAT3 pathways. The inhibition of ERK1/2 demonstrates a significant reduction in the IL-17-mediated STAT3 phosphorylation, suggesting a functional role of the ERK1/2-JAK2/STAT3 crosstalk [65].

The ataxia-telangiectasia mutated (ATM) kinase is a DNA damage-inducible kinase that phosphorylates various substrates participating in cell cycle regulation and DNA repair [66]. It stabilizes P53 after DNA damage by phosphorylating multiple residues near the ring domain of MDM2, including the Ser429 residue [67]. This modification of MDM2 causes its autoubiquitination and degradation. Others reported that ATM activates checkpoint kinase 2 (Chk2), which phosphorylates P53 on Ser20 residue. The phosphorylation of P53 disrupts its binding to the MDM2 [68]. The ataxia-telangiectasia and Rad3 related (ATR) kinase is closely related to ATM. It also regulates the cell cycle checkpoint kinases and phosphorylates P53 at Ser15 and Ser37 residues. Inhibition of ATR shows promising results in cancer therapy [69]. Inflammation enhances the production of reactive oxygen species (ROS) and reactive nitrogen species (RNS), resulting in cellular DNA damage through the formation of 8-oxo-7,8-dihydro-2′-deoxyguanosine and 8-nitroguanine [70]. AUY-922 suppresses the LPS-induced activation of ATM and ATR kinases in lung tissues which indicates its potential to protect against DNA damage during inflammation. Previous studies reported the inhibition of ATM expression by the Hsp90 inhibitor 17-DMAG in non-small cell lung cancer cell lines [71].

The inhibition of Hsp90 protein by AUY-922 induces the levels of pIRE1α in lung tissues. IRE1α exhibits endoribonuclease activity which regulates the activation of X-box binding protein-1 (XBP1) by the cleavage of XBP1 mRNA to form spliced XBP1 (XBP1s). The spliced XBP1 is required for ER expansion and increases a subset of UPR target genes involved in ER proteostasis. Deletion of XBP1 in intestinal epithelial cells induces inflammation and develops inflammatory bowel disease (IBD) [72]. In addition, IRE1α RNase cleaves the ER-associated mRNAs or non-coding functional RNAs which causes their degradation through an IRE1-dependent decay (RIDD). It also modulates the protein folding load, inflammation and inflammasome-related signaling pathways [73]. IRE1α cleaves miRNAs that are functionally involved in metabolism and inflammation in the liver. This modification causes the degradation of miRNAs [74].

The correlation between UPR and inflammation has been demonstrated in many diseases including obesity. The obesity-related chronic inflammation disrupts IRE1α by increasing nitric oxide synthase (iNOS) activity, which in turn causes S-nitrosylation of IRE1α. Those events lead to the reduction of hepatic IRE1α-mediated XBP1 splicing and, subsequently, the disruption of glucose homeostasis. The restoration of the IRE1α-mediated XBP1 splicing improves glucose homeostasis [75]. In addition to the canonical role as a UPR mediator, IRE1α is associated with other cellular functions such as cell differentiation, angiogenesis and metabolism. IRE1α physically interacts with filamin A, a crucial factor of actin crosslinking, through its proline-rich region at the distal C-terminal. Stimulation of IRE1α phosphorylates filamin A and upregulates Rac1 activity and hence, modulates the cytoskeleton remodeling [76].

An emerging body of evidence suggests that Hsp90 is necessary for the stability and function of IRE1α [77]. Cdc37, a co-chaperone of Hsp90, directly binds with IRE1α via a highly conserved cytosolic motif of IRE1α. Knockdown of Cdc37, or breakdown of the Cdc37 and IRE1α interaction causes significant induction of basal IRE1α activity in INS-1 cells [78]. 17-AAG activates the UPR pathway by inducing the activation of IRE1α in myeloma plasma cells [79]. Geldanamycin promotes the dissociation of BiP from IREα and induces IRE1α activation [77]. Our study supports our ongoing research on the development of new therapeutics based on Hsp90 inhibition against lung inflammatory diseases. Since AUY-922 induces the anti-inflammatory IRE1α (Figure 3D), it is possible that this UPR sensor may exert a pivotal role in the protective activities of Hsp90 inhibitors in the vasculature.

## 4. Materials and Methods

### 4.1. Reagents

AUY-922 (101756-820), anti-mouse IgG HRP-linked antibody (95017-554), anti-rabbit IgG HRP-linked whole (95017-556), nitrocellulose membranes (10063-173), RIPA buffer (AAJ63306-AP) and EZBlock^TM^ block protease inhibitor cocktail (10190-060) were obtained from VWR (Radnor, PA, USA). APE1/Ref1 (4128S), phospho-p44/42 MAPK (Erk1/2) (9101S), p44/42 MAPK (Erk1/2) (9102S), phospho-JAK2 (3776S), JAK2 (3230S), phospho-STAT3 (9145S), STAT3 (4904S), phospho-ATR (2853S), ATR (2790S), phospho-ATM(13050S), ATM (2873S), phospho-AMPK (2535S), AMPK (2793S), IL1-β (12703S), IL-6 (12153S), IRE1α (3294S), phospho-P38 (9211S), P38 (9212S), MCP-1 (2027S) and TNFα (3707S) antibodies were purchased from Cell Signaling (Danvers, MA, USA). Lipopolysaccharides (LPS) (L4130) and β-actin antibodies (A5441) were purchased from Sigma-Aldrich (St Louis, MO, USA). The phospho-IRE1α (PA1-16927) antibody was purchased from Thermo Scientific (Rockford, IL, USA).

### 4.2. Animals

Seven-week-old C57BL/6 (male) mice were purchased from Envigo (Indianapolis, IN). They were maintained in a 12:12-h light/dark cycle, in pathogen-free conditions. The temperature was controlled (22–24 °C), as well as the humidity (50–60%). All experimental procedures were approved by the University of Louisiana Monroe Institutional Animal Care and Use Committee (IACUC) (19JUN-NB-01) and were in line with the principles of human animal care adopted by the American Physiological Society.

### 4.3. In Vivo Treatments

Stock solutions of *E. coli* LPS (0111:B4) and the Hsp90 inhibitor AUY-922 were prepared in saline and 10% DMSO, respectively [41,49,80]. The in vivo treatment dose and timing for the LPS and AUY-922 were determined as per the previous studies in C57BL/6 mice [49,81]. Mice received vehicle (saline) or LPS (1.6 mg/Kg) via intra-tracheal instillation. After 24 h of LPS administration, mice received AUY-922 (10 mg/kg) intraperitoneally and were sacrificed 48 h later.

### 4.4. Collection of BALF and Total Protein Measurement

Bronchoalveolar lavage fluid (BALF) from mouse lungs was obtained by instilling and withdrawing 1ml of PBS by using a tracheal cannula. The total protein concentration in BALF was estimated with the bicinchoninic acid protein assay kit (Thermo Scientific, Rockford, IL, USA).

### 4.5. Western Blot Analysis

RIPA buffer and EZBlock^TM^ block protease inhibitor cocktail were used to isolate the proteins from homogenized lung tissues. The protein concentration was measured using the BCA protein assay method. Proteins were separated according to their molecular weight by electrophoresis onto sodium dodecyl sulfate (SDS-PAGE) Tris-HCl gels. A wet transfer technique was used to transfer the proteins onto nitrocellulose membranes. All the membranes were incubated for 1 h at room temperature in a solution of 5% non-fat dry milk and subsequently, exposed to appropriate primary antibodies (1:1000) at 4 °C overnight. The following day, membranes were incubated with the corresponding HRP-linked secondary antibodies (1:2000). Protein bands were detected by the SuperSignal™ West Pico PLUS chemiluminescent substrate (PI34578). The images of the protein bands were captured in a ChemiDoc™ Touch Imaging System from Bio-Rad (Hercules, CA, USA). β-actin was the loading control unless stated otherwise in the densitometry graph. All reagents were obtained from VWR (Radnor, PA, USA).

### 4.6. Densitometry and Statistical Analysis

Image J software (NIH) was utilized to perform the densitometry of the immunoblots. All data are expressed as mean values ± SEM (standard error of the mean). The Student’s t-test was used to determine statistically significant differences in protein expression among the groups. Bodyweight and BALF protein concentration data were analyzed by one-way ANOVA with the post hoc Dunnett test for the comparison of the treatment groups and the control group. A value of *p* < 0.05 was considered significant. GraphPad Prism (version 5.01) was used to analyze the data. The letter n represents the number of experimental repeats.

## 5. Conclusions

Our work substantiates our ongoing efforts on the elucidation of the mechanisms involved in the protective activities of Hsp90 inhibitors in inflamed tissues, and suggest that those compounds may deliver novel therapeutic possibilities against lung inflammatory diseases and sepsis.

## Data Availability

The data presented in this study are available on request from the corresponding author.
